# Diffusion Basis Spectrum and Diffusion Tensor Imaging Detect Hippocampal Inflammation and Dendritic Injury in a Virus-Induced Mouse Model of Epilepsy

**DOI:** 10.3389/fnins.2018.00077

**Published:** 2018-02-15

**Authors:** Jie Zhan, Tsen-Hsuan Lin, Jane E. Libbey, Peng Sun, Zezhong Ye, Chunyu Song, Michael Wallendorf, Honghan Gong, Robert S. Fujinami, Sheng-Kwei Song

**Affiliations:** ^1^Department of Radiology, the First Affiliated Hospital, Nanchang University, Jiangxi, China; ^2^Department of Radiology, Washington University in St. Louis, St. Louis, MO, United States; ^3^Department of Pathology, University of Utah, Salt Lake City, UT, United States; ^4^Department of Chemistry, Washington University in St. Louis, St. Louis, MO, United States; ^5^Department of Biomedical Engineering, Washington University, St. Louis, MO, United States; ^6^Department of Biostatistics, Washington University in St. Louis, St. Louis, MO, United States; ^7^Hope Center for Neurological Disorders, Washington University School of Medicine, Washington University in St. Louis, St. Louis, MO, United States

**Keywords:** seizures, Theiler's murine encephalomyelitis virus, diffusion basis spectrum imaging, diffusion tensor imaging, hippocampal CA1 region, dendrite injury, inflammation, magnetic resonance imaging

## Abstract

Hippocampal CA1 inflammation and dendritic loss are common in epilepsy. Quantitative detection of coexisting brain inflammation and injury could be beneficial in monitoring disease progression and assessing therapeutic efficacy. In this work, we used conventional diffusion tensor imaging (DTI, known to detect axonal injury and demyelination) and a novel diffusion basis spectrum imaging (DBSI, known to detect axonal injury, demyelination, and inflammation) to detect hippocampal CA1 lesions resulting from neuronal dendritic injury/loss and concomitant inflammation in Theiler's murine encephalomyelitis virus (TMEV)-induced seizure mice. Following the cross-sectional *ex vivo* diffusion magnetic resonance imaging measurements, immunohistochemistry was performed to validate DTI and DBSI findings. Both DTI and DBSI detected immunohistochemistry-confirmed dendritic injury in the hippocampal CA1 region. Additionally, DBSI-derived restricted isotropic diffusion tensor fraction correlated with 4',6-diamidine-2'-phenylindole dihydrochloride (DAPI)-positive nucleus counts, and DBSI-derived fiber fraction correlated with dendrite density assessed by microtubule-associated protein 2 staining. DTI-derived fractional anisotropy (FA) correlated with dendrite density and negatively correlated with DAPI-positive nucleus counts. Although both DTI and DBSI detected hippocampal injury/inflammation, DTI-FA was less specific than DBSI-derived pathological metrics for hippocampal CA1 dendritic injury and inflammation in TMEV-induced seizure mice.

## Introduction

Acute seizures, which can be initiated by various infective agents such as parasites, bacteria and viruses, are followed by a latent period leading up to the development of late unprovoked epileptic seizures (Getts et al., 2008; Vezzani et al., 2016). Early seizures generally occur within 1–2 weeks post-infection, and are reported to be risk factors for high mortality and morbidity, including the development of late spontaneous seizures (Singhi, 2011). Although the exact mechanisms underlying seizures remain to be established, inflammation cascade seems to play a critical role (Singhi, 2011; Vezzani et al., 2016). A Theiler's murine encephalomyelitis virus (TMEV)-induced seizure model has recently been developed in C57BL/6J mice to mimic infection-induced temporal lobe epilepsy (Libbey et al., 2008; Kirkman et al., 2010; Stewart et al., 2010a,b; Libbey and Fujinami, 2011). In this model, acute seizures occur between 3 and 10 days after infection, followed by a variable latent period after which spontaneous seizures develop. Hippocampal CA1 neuronal death and dendritic injury/loss are the hallmark pathologies in TMEV-induced seizure mice. TMEV-infected mouse brains exhibit various signs of neuroinflammation during acute seizures, including activated microglia/macrophages, gliosis, perivascular cuffing, and increased mRNA expression of proinflammatory cytokines, supporting the key role that inflammation plays in the development of acute seizures in the TMEV-induced seizure model (Kirkman et al., 2010; Libbey et al., 2011; Cusick et al., 2013).

The hippocampus, important for learning and memory formation, is particularly vulnerable to epilepsy and is highly associated with inflammation (Lado et al., 2002). Quantification of hippocampal inflammation and injury could not only accurately detect the underlying pathologies but also reflect the disease progression in epilepsy. Diffusion tensor imaging (DTI) has been widely used to noninvasively assess white matter integrity in central nervous system (CNS) diseases (Song et al., 2002, 2003). It has been successfully applied in epilepsy patients to assess the evolution of axonal damage after corpus callosotomy (Concha et al., 2006). The highly organized hippocampal structure, i.e., densely packed neurons surrounded by layers of dendrites and axons (Shepherd et al., 2006), is also uniquely suited for DTI examination. In rats with status epilepticus induced via kainic acid or pilocarpine administration, DTI-derived fractional anisotropy (FA) of the dentate gyrus correlated with the density of mossy fibers and myelinated axons (Laitinen et al., 2010). Despite being an effective marker of neuronal pathologies, decreased FA in epilepsy may be caused by neuronal injury/loss and/or inflammation. Thus, DTI pathological metrics in epilepsy may be sensitive to hippocampal injuries although not necessarily specific.

We have recently developed a novel diffusion basis spectrum imaging (DBSI) method to resolve crossing fiber tracts, to distinguish and quantify cerebrospinal fluid partial volume effects, and to quantitatively assess axonal injury, demyelination, and inflammation in CNS diseases (Wang et al., 2011, 2015; Chiang et al., 2014). In the current study, we examined postmortem perfusion-fixed brains from mice with TMEV-induced seizures to determine whether DBSI, a model initially developed to address white matter structure and pathologies, is amenable to detect hippocampal CA1 neuronal dendritic injury/loss in the presence of infiltrating inflammatory cells in the acute stage of viral infection at the peak of seizures.

## Materials and methods

### Animals

Four- to five-week-old male C57BL/6J mice (Jackson Laboratories, Bar Harbor, ME, USA) were used for this study. Animals (5 mice per group) were kept on a 12-h light-dark cycle with free access to food and water. This study was carried out in accordance with the recommendations of the Committee on Care and Use of Laboratory Animals, Institute of Laboratory Animals Resources, National Research Council. The protocol was approved by the Institutional Animal Care and Use Committee of the University of Utah. This study complies fully with the ARRIVE guidelines.

### Infection of mice and seizure monitoring

Mice were randomly divided into two groups and intracerebrally injected with 3 × 10^4^ plaque forming units of the Daniels (DA) strain of TMEV or 20 μl of phosphate-buffered saline (PBS) alone (sham) under anesthesia, as previously described (Libbey et al., 2008). The site of injection was in the posterior parietal cortex of the right cerebral hemisphere to a depth of 2 mm [posterior (caudal) and medial of the right eye at approximately begma−2 mm and interaural +8 mm] (Kirkman et al., 2010). The needle had a William's collar to limit penetration of the tip to 2 mm. To monitor seizure activity, mice were observed for 1 h/day for days 3 through 6 following infection. Seizure activity was graded using the Racine scale as follows: stage 1, mouth and facial movements; stage 2, head nodding; stage 3, forelimb clonus; stage 4, rearing; and stage 5, rearing and falling (Racine, 1972). Seizure burden was analyzed by assessing both seizure incidence (number of mice having seizures, numbers of observed seizures per mouse) and seizure severity (cumulative seizure score per mouse, numbers of seizures scored as stage 5). Only TMEV-infected mice that had acute behavioral seizures were imaged, while mice from the TMEV group that were not experiencing seizures were excluded from the study. Six days after DA virus (*n* = 5) or PBS (*n* = 5) injection, mice were perfusion-fixed with 4% paraformaldehyde in PBS. Brain specimens were left in 4% paraformaldehyde for overnight at 4°C then transferred to PBS. Samples were shipped overnight to Washington University in St. Louis, MO, USA for *ex vivo* DBSI and immunohistochemical (IHC) analysis.

### Magnetic resonance imaging (MRI) measurements

Upon arrival in St. Louis, the fixed brains were removed from the transport tubes and placed in 5-ml syringes containing fresh PBS. Air bubbles were removed to prevent susceptibility artifacts. The syringes containing brain specimens were placed inside a solenoid coil made of copper foil (17 mm in diameter and 27 mm in length). Diffusion weighted MRI data were acquired on an 11.74-T Agilent DirectDrive™ small-animal MRI scanner (Agilent technologies, Santa Clara, CA, USA), equipped with Magnex/Agilent HD imaging gradient coil (Magnex/Agilent, Oxford, UK) with pulse gradient strength up to 120 G/cm and a gradient rise time ≤ 270 μs. After obtaining T1- and diffusion weighted scout images, a diffusion weighted, multi-echo spin-echo imaging sequence was performed with a 99-direction diffusion-encoding scheme to acquire diffusion weighted images of 25 contiguous coronal slices covering the whole mouse brain. Acquisition parameters were: repetition time (TR) = 3,000 ms, echo time (TE) = 32.8 ms, Δ = 18 ms, δ = 5 ms, field-of-view (FOV) = 19.2 × 19.2 mm^2^, matrix size = 192 × 192, slice thickness = 0.5 mm. The max *b*-value = 3,030 s/mm^2^. Total data acquisition time was 16 h.

### Diffusion basis spectrum imaging

Diffusion weighted MRI data were analyzed with both DBSI and conventional DTI analysis packages developed in-house with Matlab ((Wang et al., 2011, 2015; Chiang et al., 2014; Cross and Song, 2017); MathWorks, Natick, MA, USA). In the current study, we used a single anisotropic tensor to model the dendrites in the CA1 region, and a spectrum of isotropic diffusion tensors to estimate the extent of inflammation associated cell infiltration and vasogenic edema, and neuronal loss. Diffusion weighted data were analyzed according to Equation (1):

(1) $$\begin{array}{l} S_{k}=fe^{-\left|\vec{b_{k}}\right|\lambda_{⊥}}e^{-\left|\vec{b_{k}}\right|(\lambda_{∥}-\lambda_{⊥}){\text{cos}}^{2}\Phi_{k}}+\int_{a}^{b}f\left(D\right)e^{-\left|\vec{b_{k}}\right|D}dD\\ \;\left(k=1,\;2,\;3,\ldots ,\;99\right). \end{array}$$

The quantities *S*_*k*_ and $\left|\vec{b_{k}}\right|$ are the signal and *b*-value of the *k*^*th*^ diffusion gradient, Φ_*k*_ is the angle between the *k*^*th*^ diffusion gradient and the principal direction of the anisotropic tensor, λ_||_ and λ_⊥_ are the axial and radial diffusivities of the anisotropic tensor, *f* is the signal intensity fraction for the anisotropic tensor, and *a* and *b* are the low and high diffusivity limits for the isotropic diffusion spectrum *f*(*D*), reflecting cellularity and edema, respectively. DBSI derived *f* represents dendritic density (fiber fraction) in the image voxel. The isotropic diffusion spectrum was tentatively divided into components based on ADC values: the restricted isotropic diffusion (0 ≤ ADC ≤ 0.6 μm^2^/ms) fraction reflects cellularity, the hindered isotropic diffusion (0.6 < ADC ≤ 2 μm^2^/ms) represents vasogenic edema, and/or tissue loss (Wang et al., 2011, 2014, 2015; Chiang et al., 2014). Regions-of-interest were manually drawn on the hippocampal CA1 region, according to a previous literature study (Richards et al., 2011), on diffusion weighted images with ITK-SNAP software (Yushkevich et al., 2006) (www.itksnap.org).

### Immunohistochemistry

After MRI, brains were dissected and embedded in paraffin. The embedded tissues were sectioned using a sliding microtome at 5-μm thickness. Sections containing the hippocampal area (from Bregma−4.04 mm to−0.94 mm) were collected for analysis. The sectioned tissues were deparaffinized, rehydrated, and then blocked using 2% bovine serum albumin (Sigma Inc., St. Louis, MO, USA) and 10% normal goat-serum solution for 30 min at room temperature to prevent non-specific binding. Slides were incubated overnight at 4°C with mouse monoclonal antibody against neuronal nuclei (NeuN, 1:300 dilution, Millipore, Billerica, MA, USA) for mature neurons, mouse monoclonal antibody against microtubule-associated protein 2 (MAP2, 1:300, Sigma) for neuronal dendrites, and rabbit polyclonal antibody against ionized calcium-binding adapter molecule 1 (IBA1, 1:300, Wako, Osaka, Japan) for microglia.

After rinsing, goat anti-mouse IgG or goat anti-rabbit IgG conjugated Alexa 488 (1:240) was applied to visualize neurons, dendrites or microglia. Finally, slides were covered using Vectashield Mounting Medium with 4′,6-diamidine-2′-phenylindole dihydrochloride (DAPI, Vector Laboratory, Inc., Burlingame, CA, USA). Histological slides were examined with a Nikon Eclipse 80i fluorescence microscope equipped with a 40 × objective, and images from the center of the hippocampus were captured with a black-and-white CCD camera controlled by MetaMorph software (Universal Imaging Corporation, Sunnyvale, CA, USA). The 40 × IHC images covered the whole left or right hippocampus. Using ImageJ (http://rsbweb.nih.gov/ij/plugins/volume-viewer.html, NIH, Bethesda, MD, USA), quantification of MAP2-antibody-positive dendrites was performed on the stratum radiatum layer (apical dendrites) of the CA1 region [0.033 ± 0.013 mm^2^, mean ± standard deviation (SD)], and DAPI-positive cell nuclei were quantified over the entire hippocampal CA1 region (0.089 ± 0.026 mm^2^, mean ± SD). MAP2 images were then converted to 8-bit gray scale and subjected to background subtraction followed by bilateral filter for edge preservation, watershed segmentation, thresholding, and the analyze particles macro for MAP2 area calculation and then normalized by the stratum radiatum layer of the CA1 region (MAP2-positive area fraction). Background subtraction, watershed segmentation, threshold determination, and analyze particles were used for DAPI counts, and then normalized by the entire CA1 region (DAPI-positive nucleus density).

### Statistical analysis

Student *t*-test was used to determine whether any differences existed between the two groups and a significance level of 0.05 was used for all tests. Pearson correlation was used to test the association between IHC results and DBSI or DTI parameters (i.e., MAP2-positive area ratio and DBSI-derived fiber fraction, MAP2-positive area ratio and DTI FA, DAPI-positive nucleus density and DBSI-derived restricted isotropic diffusion fraction, DAPI-positive nucleus density and DTI FA) in the hippocampal CA1 region. All data are expressed as mean ± SD.

## Results

### Acute seizure scores in TMEV-infected mice

Seizures were scored as described in the methods for 4 days following infection (days 3 through 6) and seizure burden, both seizure incidence and seizure severity, was assessed. As can be seen in Table 1, all 5 mice in the TMEV-infected group had severe seizures, whereas none of the PBS (Sham) injected mice had any seizures.

**Table 1:** Acute seizures in TMEV-infected mice.

**Groups**	**Seizure Incidence**		**Seizure Severity**	
	**# Mice w/Seizures (%)**	**Average Observed Seizures/Mouse^a^**	**Average Cumulative Seizure Score/Mouse^a^**	**# Seizures Scored as Stage 5 (%)**
TMEV	5/5 (100)	2 ± 0	9.4 ± 0.4	8/10 (80%)
Sham	0/5 (0)	0 ± 0	0 ± 0	0 (0)

*TMEV, Theiler's murine encephalomyelitis virus*.

a *Mean ± standard error of the mean*.

### DTI metrics of the hippocampus

**Figure 1:**
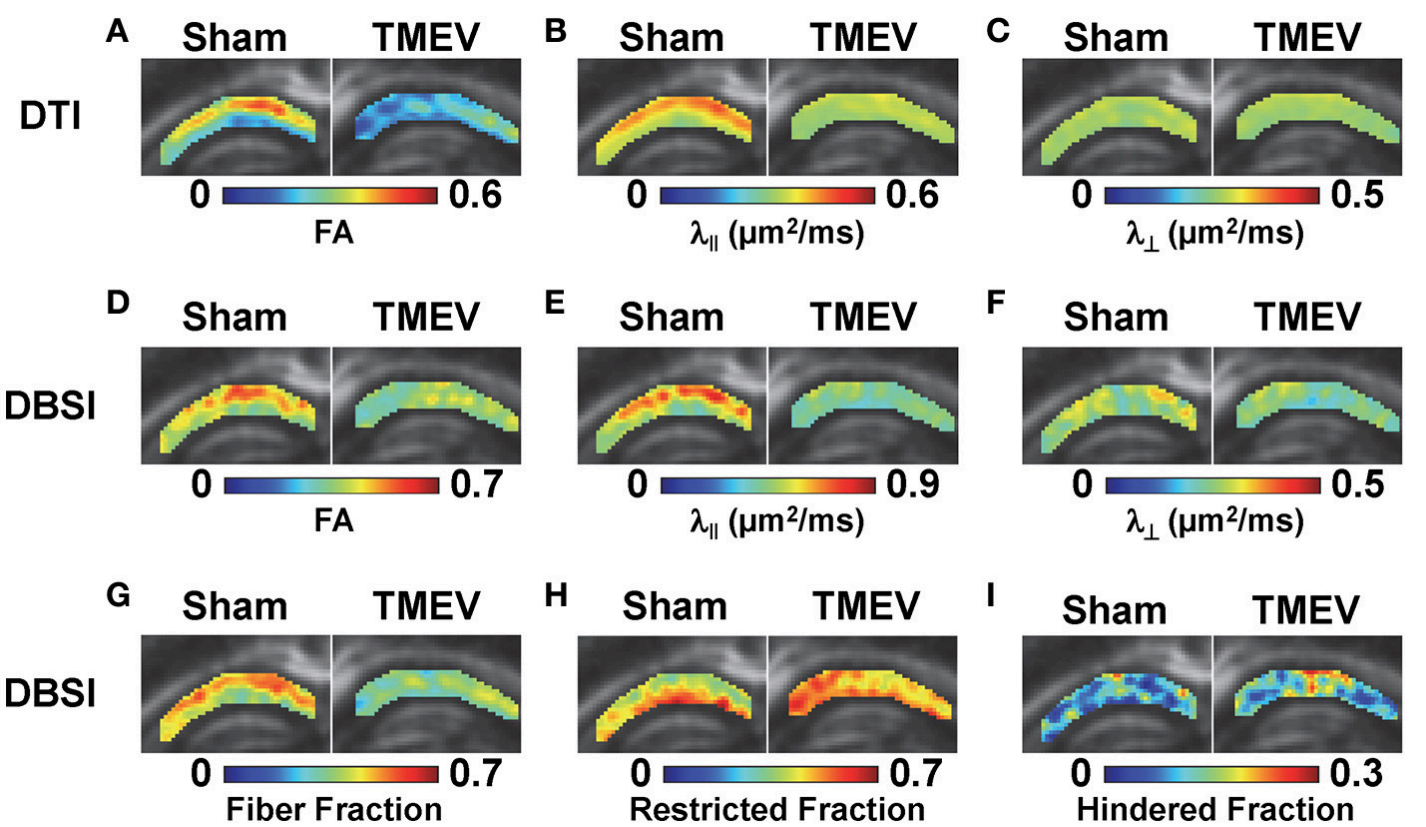
At 6 days after TMEV or sham (PBS) infection, mice were perfusion-fixed and brains were extracted for *ex vivo* diffusion basis spectrum imaging (DBSI) measurements. Color-coded hippocampal CA1 diffusion tensor imaging (DTI) and DBSI metric maps from one representative sham (left) and TMEV-infected (right) mouse were overlaid on diffusion weighted images. DTI-FA **(A)** and DTI-λ_∥_ **(B)** decreased in TMEV hippocampal CA1, suggesting dendritic injury, while no statistically significant changes in DTI-λ_⊥_ were observed **(C)**. Both anisotropic (describing properties of dendritic integrity) and isotropic (describing environment surrounding dendrites) diffusion tensors were quantified by DBSI, offering more detailed insights of the underlying pathologies responsible for the observed changes in DTI metrics. Specifically, decreased DBSI-fiber FA **(D)** and DBSI-λ_⊥_ **(F)** may reflect the structural change (dendritic swelling and inflammatory cell infiltration) in CA1 resulting from dendritic injury and loss; decreased DBSI-λ_∥_ **(E)** suggests injury of residual dendrites; decreased DBSI-fiber fraction **(G)** estimates dendritic loss; increased DBSI-restricted isotropic diffusion tensor fraction **(H)** quantifies increased cellularity, and the increased DBSI-hindered isotropic diffusion tensor fraction **(I)** may reflect the increased extent of edema or structural changes in CA1 after seizures. The multiple pathological components derived by DBSI suggest confounding factors complicate the interpretation of changes seen in DTI metrics.

**Figure 2:**
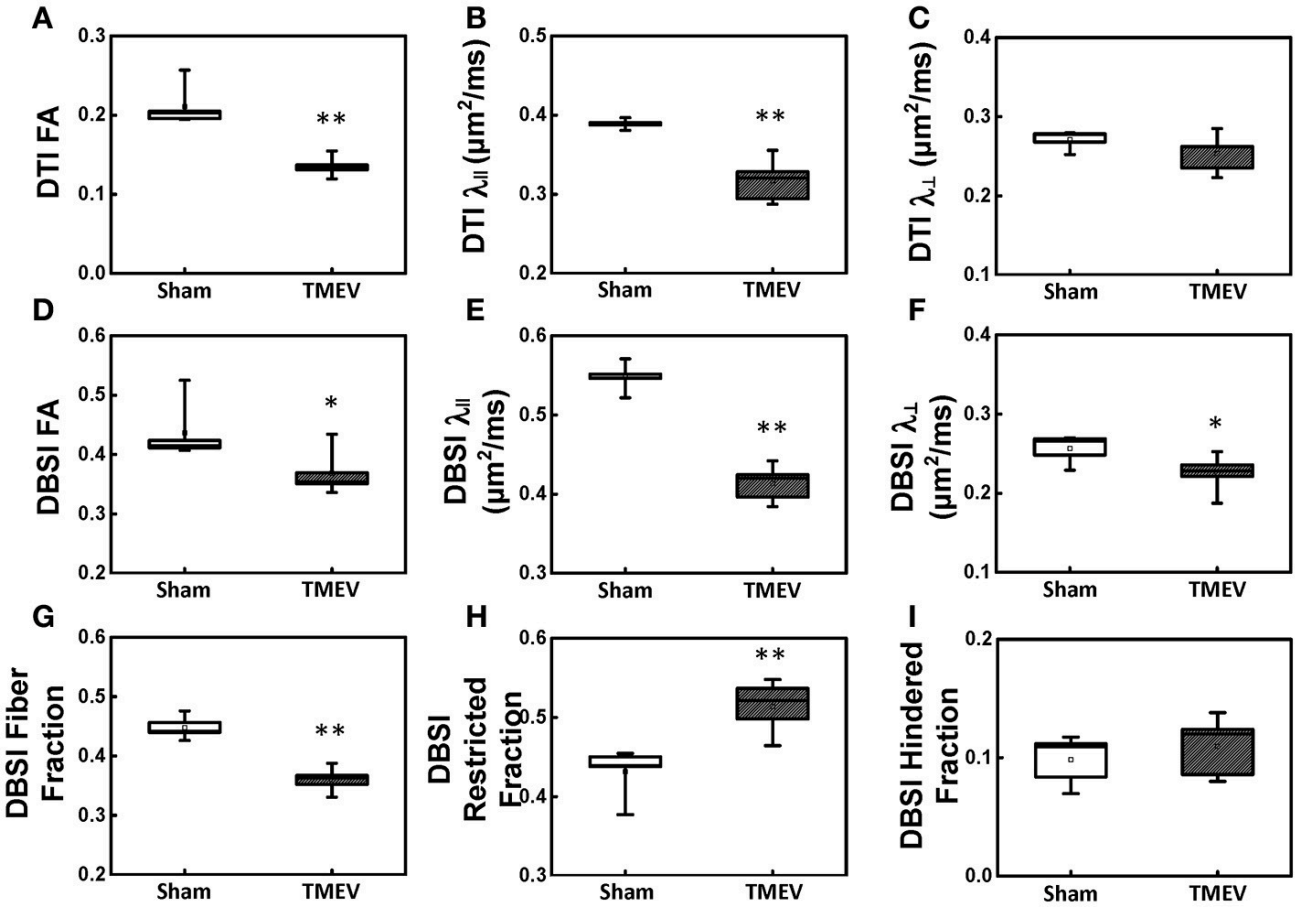
Box plots of hippocampal CA1 quantitatively summarize the changes in diffusion MRI metrics. A statistically significant 36% and 18% decrease was seen in DTI-FA (**A**, *p* < 0.005) and DTI-λ_∥_ (**B**, *p* < 0.005) in TMEV-infected hippocampal CA1 comparing with that of the sham (PBS). No statistically significant difference in DTI-λ_⊥_ was observed (**C**, *p* > 0.1). In contrast to DTI-FA, a smaller statistically significant decrease (15%) in DBSI-FA was also observed in the TMEV-infected group (**D**, *p* < 0.05). A significant 24% and 12% decrease in TMEV-infected CA1 DBSI-λ_∥_ (**E**, *p* < 0.005) and DBSI-λ_⊥_ (**F**, *p* < 0.05) was also seen. DBSI is able to assess structural and pathological changes in the environment surrounding dendrites in the hippocampal CA1 region. To this end, a statistically significant 20% DBSI-fiber fraction decrease (**G**, *p* < 0.0001; putative dendritic density) was seen along with a statistically significant 23% increase in DBSI-restricted isotropic diffusion fraction (**H**, *p* < 0.005; reflecting increased cellularity) and a statistically insignificant 12% increase in DBSI-hindered isotropic diffusion tensor fraction (**I**, *p* > 0.1). ^*^ indicates *p* < 0.05; ^**^ indicates *p* < 0.005.

Color-coded diffusion metric maps of left and right hippocampal CA1 regions were overlaid on the diffusion-weighted MRI (Figure 1). At 6 days after infection, hippocampal CA1 DTI-FA (Figure 1A) and DTI-λ_∥_ (Figure 1B) decreased in TMEV-infected mice compared to the sham control. No significant change in DTI-λ_⊥_ was observed in TMEV-infected mice compared to controls (Figure 1C). Group analysis showed that TMEV-infected mice exhibited a statistically significant 36% DTI-FA decrease (Figure 2A, *p* = 0.0004) and 18% DTI-λ_∥_ decrease (Figure 2B, *p* = 0.0004) compared to sham mice in the hippocampal CA1 region. A statistically insignificant 6% decrease in DTI-λ_⊥_ was observed (Figure 2C, *p* = 0.18).

### DBSI metrics of the hippocampus

Compared to the sham (PBS) control, TMEV-infected mice showed decreased DBSI-derived fiber FA (Figure 1D), DBSI-λ_||_ (Figure 1E), DBSI-λ_⊥_ (Figure 1F) and fiber fraction (Figure 1G), while increased DBSI-restricted fraction (Figure 1H) and DBSI-hindered fraction (Figure 1I) in the CA1 region were also observed. Group analysis further showed that TMEV-infected mice had a statistically significant 15% DBSI-FA decrease (Figure 2D, *p* = 0.044), 24% DBSI-λ_∥_ decrease (Figure 2E, *p* = 0.0001), 12% DBSI-λ_⊥_ decrease (Figure 2F, *p* = 0.047), and 20% DBSI-fiber fraction decrease (Figure 2G, *p* = 0.0001) in the CA1 region. In addition, a substantial 23% increase in restricted isotropic diffusion fraction (Figure 2H, *p* = 0.0039) and a statistically insignificant 12% increase in hindered isotropic diffusion fraction (Figure 2I, *p* = 0.47) were observed in the CA1 region in TMEV-infected mice.

### Immunohistochemistry

**Figure 3:**
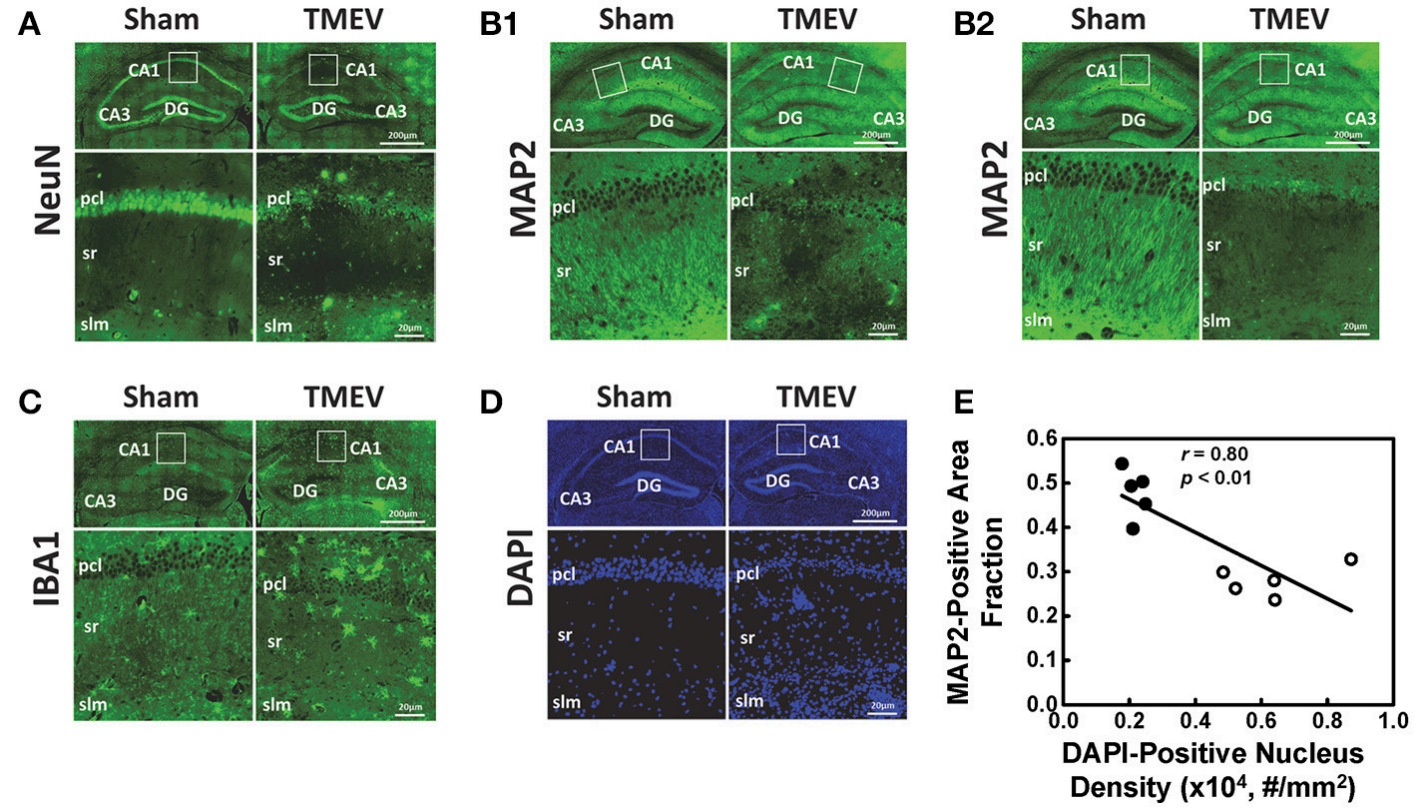
Immunohistochemical staining was performed on all tissues after MRI measurements. Representative staining results from the hippocampal CA1 regions of TMEV-infected and sham (PBS) mice were presented in 40× full and expanded views. Pyramidal cell layer neurons were stained using NeuN antibody **(A)**, revealing the significant loss of pyramidal cells in the CA1 of TMEV-infected mice. Dendrites were stained using MAP2 antibody **(B)**. Dendritic damage in the CA1 is heterogeneous in TMEV-infected mice with regions exhibiting disorganized debris **(B1)** and regions of total loss of dendrites **(B2)**. Activated microglia were stained using IBA1 antibody **(C)**, and an apparent increase in numbers of activated microglia was seen in TMEV-infected mice. The change in the CA1 region cellularity was assessed by examining DAPI **(D)** staining, where increased DAPI-positive staining was seen in TMEV-infected mice. Notably, the MAP2-positive staining area fraction negatively correlated with the DAPI-positive nucleus density (**E**, filled symbols = sham; open symbols = TMEV).

Representative IHC staining results from the hippocampal CA1 regions of TMEV-infected and sham mice are presented in 40 × full and expanded views (Figure 3). Similar to a previous literature report, TMEV-infected mice showed significant neuronal death in the hippocampus (Stewart et al., 2010b), especially the pyramidal cell layer of the CA1 region (NeuN, Figure 3A), followed by substantial apical dendritic loss (MAP2, Figure 3B1) and injury (MAP2, Figure 3B2) in the stratum radiatum layer of the CA1 region. Activated microglia (IBA1, Figure 3C) and increased cellularity (DAPI, Figure 3D) were detected in the entire CA1 region, suggesting inflammatory cell infiltration. The MAP2-positive staining area fraction negatively correlated with the DAPI-positive nucleus density (*r* = 0.08, *p* < 0.01, Figure 3E).

### Correlation between diffusion MRI findings and IHC

**Figure 4:**
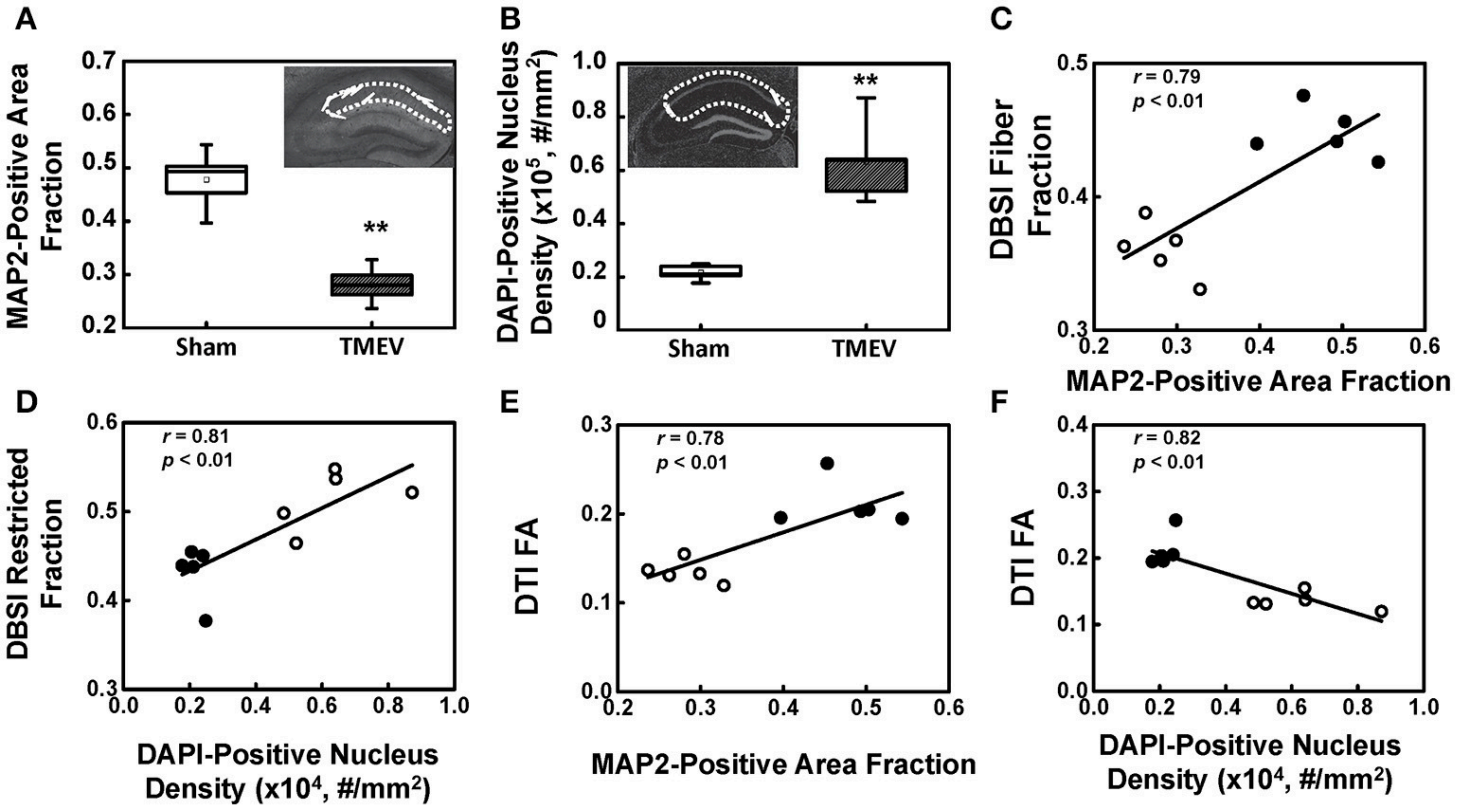
MAP2 and DAPI immunohistochemical staining quantified in the region-of-interest outlined with dotted lines in the inset of **(A**,**B)**. MAP2-positive area fraction significantly decreased in the CA1 of TMEV-infected mice comparing with that of the sham (PBS) (**A**, *p* < 0.005). In contrast, DAPI-positive nuclei density significantly increased in the CA1 of TMEV-infected mice (**B**, *p* < 0.005). A regression analysis of DBSI-metrics and immunohistochemical staining was performed to demonstrate the presence of a linear relationship between MAP2-positive area fraction and DBSI-derived fiber fraction **(C)**, and DAPI density and DBSI-derived restricted fraction **(D)**. DTI-FA correlated with MAP2-positive area fraction **(E)**, and negatively correlated with the DAPI-positive nucleus density **(F)**. ^**^ indicates *p* < 0.005.

There was a 41% decrease in MAP2-positive (dendrites) area fraction in the stratum radiatum layer of the CA1 region (Figure 4A), and a 192% increase in DAPI-positive nucleus density in the entire CA1 region (Figure 4B). DBSI-derived fiber fraction correlated with MAP2-positive area fraction (*r* = 0.79, *p* < 0.01, Figure 4C), and the DBSI-derived restricted fraction correlated with the DAPI-positive nucleus density (*r* = 0.81, *p* < 0.01, Figure 4D). DTI FA correlated with MAP2-positive area fraction (*r* = 0.78, *p* < 0.01, Figure 4E), and negatively correlated with the DAPI-positive nucleus density (*r* = 0.82, *p* < 0.01, Figure 4F).

## Discussion

Hippocampal damage is common in seizures and epilepsy. DeGiorgio and colleagues (DeGiorgio et al., 1992) conducted a pilot study of the hippocampus in patients with severe status epilepticus and detected significantly reduced neuronal densities in the CA1 and CA3 regions. Hippocampal sclerosis, characterized by cell loss and gliosis, is the most common feature of mesial temporal lobe epilepsy (Thom, 2014). Growing evidence shows hippocampal damage varying with seizure severity or frequency, ranging from gliosis and a small reduction in neuron number to frank necrosis with destruction of the hippocampus (Lothman and Collins, 1981). Noninvasive MRI approaches have seen increased applications in detecting epileptic hippocampal pathologies. Conventional T2-weighted MRI has shown time-dependent hippocampal hyperintensities in seizure mice (Roch et al., 2002; Buenz et al., 2009). Volumetric MRI techniques have been widely applied to analyze the volumetric changes in epilepsy. Several articles have reported reliable detection of hippocampal atrophy in patients with temporal lobe epilepsy based on volumetric MRI as a surrogate marker of hippocampal sclerosis (Jack et al., 1990; Jackson et al., 1990; Jack, 1994).

The well-organized structure of the hippocampus makes it ideal to detect hippocampal injury using diffusion MRI (Shepherd et al., 2006). Assaf et al. (2003) applied DTI in patients with unilateral temporal lobe epilepsy and detected significantly increased mean diffusivity and decreased FA in the hippocampus ipsilateral to the seizure focus, as compared with those in the contralateral side. In our study, we examined hippocampal CA1 in TMEV-induced seizure mice at 6 days after infection using *ex vivo* diffusion MRI and IHC. Both DTI- and DBSI-derived λ_∥_ decreased in TMEV-infected mice (Figures 2B,E), suggesting dendritic injury. Decreased DBSI-fiber FA (Figure 2D) and DBSI-λ_⊥_ (Figure 2F) may reflect the structural change (dendritic swelling and inflammatory cell infiltration) in the CA1 region during seizures. DBSI detected mixed hippocampal pathologies including dendritic injury/loss (decrease fiber FA and fiber fraction, Figures 2D,G), and inflammation (increased cellularity and vasogenic edema, Figures 2H,I). Both DTI-FA and DBSI-fiber FA reflect dendritic damage. The more pronounced decrease in DTI-FA (Figure 2A) than DBSI-fiber FA (Figure 2D) may be a result of confounding effects of inflammatory cell infiltration (Figure 2H), and vasogenic edema or tissue loss (Figure 2I). Post-MRI MAP2 staining revealed disorganized dendritic debris and dendritic loss (Figure 3B) in the CA1 region. The decreased anisotropic diffusion tensor fraction (fiber fraction), and increased restricted isotropic diffusion tensor fraction (cellularity), correlated with dendritic loss and increased DAPI-positive nucleus density in the CA1 region as assessed by IHC (Figures 4C,D), respectively. Therefore, decreased DTI-FA may result from increased/decreased cellularity and/or decreased dendritic density. Thus, DTI-FA would be sensitive to dendritic injury and/or increased/decreased cellularity. In contrast, DBSI-fiber fraction directly reflects dendritic density while DBSI-restricted fraction only reflects cellularity changes.

Accumulating evidence supports that hippocampal damage in epilepsy may be related to inflammation (Aronica and Crino, 2011). In patients with temporal lobe epilepsy with hippocampal sclerosis, prominent activation of microglia/macrophages and astrogliosis has been shown in the hippocampus (Aronica and Gorter, 2007; Ravizza et al., 2008). Infection of the CNS is one of the inciting events of acquired epilepsies (Hauser et al., 1996; Pitkänen et al., 2007). Neuronal excitability following inflammation cascade is deemed a common mechanism for early seizures after CNS infection (Choi and Koh, 2008; Vezzani et al., 2011). In the TMEV-induced seizure mice, the innate immune response was implicated as contributing to the development of acute seizures and relates to hippocampal damage (Kirkman et al., 2010). CNS infection activates microglia which release proinflammatory cytokines (tumor necrosis factor-α and interleukin-6) that modulate glutamate homeostasis. The excessive extracellular glutamate level is excitotoxic leading to seizures and epilepsy (Choi and Koh, 2008).

An unmet need in epilepsy is to define noninvasive biomarkers that allow a longitudinal assessment of the extent of inflammation to stratify anti-inflammatory therapies for patients. Dynamic contrast enhanced MRI has been commonly used to assess blood-brain barrier breakdown after seizures in both clinical and preclinical studies (Marchi et al., 2012). However, it remains unclear to what extent vascular permeability reflects brain inflammation. Positron emission tomography (PET) ligands targeting the 18 kDa translocator protein, which is overexpressed on activated microglia and reactive astrocytes, detect neuroinflammation in seizure foci (Banati, 2002; Kumar et al., 2008). However, the effectiveness of this PET marker remains to be seen (Banati et al., 2000; Xie et al., 2012). Although increased myoinositol content measured by magnetic resonance spectroscopy has been used to monitor and quantify the degree of astrocytic activation in specific brain regions (Turkdogan-Sozuer et al., 2000; Hammen et al., 2008), the myoinositol signal may be diluted by inflammation-induced edema. Current results demonstrate the unique utility of DBSI in detecting inflammation as increased restricted isotropic diffusion tensor fraction (reflecting increased cellularity, Figure 2H) and slightly increased hindered isotropic diffusion tensor fraction (reflecting vasogenic edema, Figure 2I) in the hippocampal CA1 region from TMEV-induced seizure mice. Increased DAPI-positive nucleus counts coupled with decreased NeuN-positive staining suggests a significantly increased inflammatory cell infiltration in the CA1 region of TMEV-induced seizure mice, supported by the increased IBA1 staining of activated macrophages/microglia (Figures 3C,D). The current IHC findings are consistent with previous reports of TMEV-induced seizure mice (Kirkman et al., 2010).

The DBSI restricted isotropic diffusion fraction in the hippocampal CA1 region positively correlated with DAPI-positive nucleus density, supporting the previous finding that DBSI quantitatively assessed inflammation-associated cellularity increase (Wang et al., 2011, 2013; Chiang et al., 2014; Cross and Song, 2017; Lin et al., 2017). The decreased anisotropic diffusion tensor fraction, the tentative fiber fraction reflecting dendritic density in the hippocampal CA1 region, correlated positively with MAP2-positive staining, which is also consistent with previous findings that fiber density correlated with axonal density (Wang et al., 2011, 2013; Chiang et al., 2014; Cross and Song, 2017; Lin et al., 2017). However, the histological validation of the current study is imperfect given the strong negative correlation between dendritic loss (MAP2 staining) and inflammation (DAPI-positive staining) in the hippocampus of TMEV-induced seizure mice (Figure 3E). The strong positive correlation between DTI-FA and MAP2-positive staining density, and negative correlation between DTI-FA and DAPI-positive staining suggests that DTI-FA may serve as a sensitive detector of hippocampal CA1 injury in TMEV-induced seizure mice. To definitively validate the utility of DBSI to detect and quantify hippocampal inflammation in epilepsy, a time-course study capturing evolution of inflammation and dendritic injury/loss with cross-sectional histological validation is needed.

We have previously demonstrated that the *in vivo* contrast of diffusion MRI metrics between injured and intact tissues is preserved *ex vivo* (Sun et al., 2005; Wang et al., 2011, 2015; Chiang et al., 2014). Thus, the findings of this study using fixed postmortem specimens are expected to be translated to *in vivo* animal models or patients with epilepsy. In performing diffusion MRI, a low signal-to-noise ratio method, image quality is crucial in the accuracy of measurements and appropriateness of interpretations (Chiang et al., 2014). Our baseline signal-to-noise ratio of the current data was 40, above previously determined lower bound of 20, further supporting the validity of the data. The heterogeneity of DBSI/DTI metrics within each region-of-interest reflects the regional differences in response to TMEV-induced seizures in the CA1 region, indicating the sensitivity of DBSI/DTI metrics in detection of regional difference in tissue pathologies. Currently, DBSI has been applied to multiple brain regions, spinal cord, optic nerve, and tumors. Theoretically, DBSI can be applied to all brain regions or other organs. The application of DBSI to various tissues/organs is largely limited in the appropriate validations to ensure the correctness of predicting tissue pathologies.

In conclusion, we demonstrate the capability of DBSI to detect hippocampal CA1 neuronal dendritic injury/loss and inflammation in TMEV-induced seizure mice, partially supported by IHC validation. A longitudinal and cross-sectional *in vivo* DBSI assessment of this model could quantitatively validate this finding. Our findings suggest that DBSI can be applied to assess hippocampal CA1 pathology in epilepsy. Upon further validation, DBSI-derived pathological metrics could potentially reflect hippocampal CA1 injury, and be used in other inflammatory neurological diseases.

## Author contributions

Concept and experimental design: JZ, T-HL, HG, RF, and S-KS; Protocol and code development: T-HL, PS, ZY, and CS; Generation, collection, and analysis of data: JZ, T-HL, JL, PS, ZY, CS, MW, HG, RF, and S-KS; Manuscript drafting: JZ, T-HL, JL, ZY, MW, HG, and S-KS; Critical review of the manuscript: JL, PS, CS, RF, and S-KS; Manuscript approval: JZ, T-HL, JL, PS, ZY, CS, MW, HG, RF, and S-KS.

### Conflict of interest statement

S-KS is a co-founder of DxGPS and may financially benefit if the company is successful in marketing its product(s) that is/are related to this research. The other authors declare that the research was conducted in the absence of any commercial or financial relationships that could be construed as a potential conflict of interest.

## Abbreviations

CNS: central nervous system
DA: Daniels
DAPI: 4′,6-diamidine-2′-phenylindole dihydrochloride
DBSI: diffusion basis spectrum imaging
DTI: diffusion tensor imaging
FA: fractional anisotropy
IBA1: ionized calcium-binding adapter molecule 1
IHC: immunohistochemistry
MAP2: microtubule-associated protein 2
MRI: magnetic resonance imaging
NeuN: neuronal nuclei
PBS: phosphate-buffered saline
PET: positron emission tomography
SD: standard deviation
TMEV: Theiler's murine encephalomyelitis virus.
